# Supplementary material for: Overlapping cell population expression profiling and regulatory inference in C. elegans
Source: BMC Genomics. 2016 Feb 29;17:159. doi: 10.1186/s12864-016-2482-z (PMC4772325; doi:10.1186/s12864-016-2482-z)
Supplement: Additional file 13: — Web supplement. (DOC 21 kb) [file 12864_2016_2482_MOESM13_ESM.zip › sortWeb/clusters/hier.300.clusters/35.html]

Cluster 35 

## Cluster 35

### Expression

| cnd-1 rep. 1 | cnd-1 rep. 2 | cnd-1 rep. 3 | pha-4 rep. 1 | pha-4 rep. 2 | pha-4 rep. 3 | ceh-27 | ceh-36 | ceh-6 | F21D5.9 | mir-57 | mls-2 | pal-1 | pros-1 | ttx-3 | unc-130 | hlh-16 | irx-1 | ceh-6 (+) hlh-16 (+) | ceh-6 (+) hlh-16 (-) | ceh-6 (-) hlh-16 (+) | cnd-1 singlets | pha-4 singlets | 0 | 60 | 120 | 150 | 180 | 240 | 330 | 390 | 420 | 480 | 540 | 570 | 600 | 630 | 660 | NAME | Functional description |
| --- | --- | --- | --- | --- | --- | --- | --- | --- | --- | --- | --- | --- | --- | --- | --- | --- | --- | --- | --- | --- | --- | --- | --- | --- | --- | --- | --- | --- | --- | --- | --- | --- | --- | --- | --- | --- | --- | --- | --- |
|  |  |  |  |  |  |  |  |  |  |  |  |  |  |  |  |  |  |  |  |  |  |  |  |  |  |  |  |  |  |  |  |  |  |  |  |  |  | Y22D7AR.t1 |  |
|  |  |  |  |  |  |  |  |  |  |  |  |  |  |  |  |  |  |  |  |  |  |  |  |  |  |  |  |  |  |  |  |  |  |  |  |  |  | C49G7.12 |  |
|  |  |  |  |  |  |  |  |  |  |  |  |  |  |  |  |  |  |  |  |  |  |  |  |  |  |  |  |  |  |  |  |  |  |  |  |  |  | F43C1.7 |  |
|  |  |  |  |  |  |  |  |  |  |  |  |  |  |  |  |  |  |  |  |  |  |  |  |  |  |  |  |  |  |  |  |  |  |  |  |  |  | C07G1.7 |  |
|  |  |  |  |  |  |  |  |  |  |  |  |  |  |  |  |  |  |  |  |  |  |  |  |  |  |  |  |  |  |  |  |  |  |  |  |  |  | Y49G5A.1 |  |
|  |  |  |  |  |  |  |  |  |  |  |  |  |  |  |  |  |  |  |  |  |  |  |  |  |  |  |  |  |  |  |  |  |  |  |  |  |  | T23E7.5 |  |
|  |  |  |  |  |  |  |  |  |  |  |  |  |  |  |  |  |  |  |  |  |  |  |  |  |  |  |  |  |  |  |  |  |  |  |  |  |  | *lim-7* | LIM domain family |
|  |  |  |  |  |  |  |  |  |  |  |  |  |  |  |  |  |  |  |  |  |  |  |  |  |  |  |  |  |  |  |  |  |  |  |  |  |  | C01G5.7 |  |
|  |  |  |  |  |  |  |  |  |  |  |  |  |  |  |  |  |  |  |  |  |  |  |  |  |  |  |  |  |  |  |  |  |  |  |  |  |  | C02B10.3 |  |
|  |  |  |  |  |  |  |  |  |  |  |  |  |  |  |  |  |  |  |  |  |  |  |  |  |  |  |  |  |  |  |  |  |  |  |  |  |  | *cfz-2* | Caenorhabditis FriZzled homolog |
|  |  |  |  |  |  |  |  |  |  |  |  |  |  |  |  |  |  |  |  |  |  |  |  |  |  |  |  |  |  |  |  |  |  |  |  |  |  | *sox-2* | SOX (mammalian SRY box) family |
|  |  |  |  |  |  |  |  |  |  |  |  |  |  |  |  |  |  |  |  |  |  |  |  |  |  |  |  |  |  |  |  |  |  |  |  |  |  | F13H10.9 |  |
|  |  |  |  |  |  |  |  |  |  |  |  |  |  |  |  |  |  |  |  |  |  |  |  |  |  |  |  |  |  |  |  |  |  |  |  |  |  | *frpr-9* | FMRFamide Peptide Receptor family |
|  |  |  |  |  |  |  |  |  |  |  |  |  |  |  |  |  |  |  |  |  |  |  |  |  |  |  |  |  |  |  |  |  |  |  |  |  |  | Y39G8C.2 |  |
|  |  |  |  |  |  |  |  |  |  |  |  |  |  |  |  |  |  |  |  |  |  |  |  |  |  |  |  |  |  |  |  |  |  |  |  |  |  | *fkh-2* | ForKHead transcription factor family |
|  |  |  |  |  |  |  |  |  |  |  |  |  |  |  |  |  |  |  |  |  |  |  |  |  |  |  |  |  |  |  |  |  |  |  |  |  |  | *nspd-10* | Nematode Specific Peptide family, group D |
|  |  |  |  |  |  |  |  |  |  |  |  |  |  |  |  |  |  |  |  |  |  |  |  |  |  |  |  |  |  |  |  |  |  |  |  |  |  | F48G7.12 |  |
|  |  |  |  |  |  |  |  |  |  |  |  |  |  |  |  |  |  |  |  |  |  |  |  |  |  |  |  |  |  |  |  |  |  |  |  |  |  | F27B3.5 |  |
|  |  |  |  |  |  |  |  |  |  |  |  |  |  |  |  |  |  |  |  |  |  |  |  |  |  |  |  |  |  |  |  |  |  |  |  |  |  | T01G6.10 |  |
|  |  |  |  |  |  |  |  |  |  |  |  |  |  |  |  |  |  |  |  |  |  |  |  |  |  |  |  |  |  |  |  |  |  |  |  |  |  | Y75B8A.33 |  |
|  |  |  |  |  |  |  |  |  |  |  |  |  |  |  |  |  |  |  |  |  |  |  |  |  |  |  |  |  |  |  |  |  |  |  |  |  |  | Y47G6A.32 |  |
|  |  |  |  |  |  |  |  |  |  |  |  |  |  |  |  |  |  |  |  |  |  |  |  |  |  |  |  |  |  |  |  |  |  |  |  |  |  | ZK1236.9 |  |
|  |  |  |  |  |  |  |  |  |  |  |  |  |  |  |  |  |  |  |  |  |  |  |  |  |  |  |  |  |  |  |  |  |  |  |  |  |  | F25B3.4 |  |
|  |  |  |  |  |  |  |  |  |  |  |  |  |  |  |  |  |  |  |  |  |  |  |  |  |  |  |  |  |  |  |  |  |  |  |  |  |  | F25B3.5 |  |
|  |  |  |  |  |  |  |  |  |  |  |  |  |  |  |  |  |  |  |  |  |  |  |  |  |  |  |  |  |  |  |  |  |  |  |  |  |  | R05D7.3 |  |
|  |  |  |  |  |  |  |  |  |  |  |  |  |  |  |  |  |  |  |  |  |  |  |  |  |  |  |  |  |  |  |  |  |  |  |  |  |  | ZK899.7 |  |
|  |  |  |  |  |  |  |  |  |  |  |  |  |  |  |  |  |  |  |  |  |  |  |  |  |  |  |  |  |  |  |  |  |  |  |  |  |  | K12H6.12 |  |
|  |  |  |  |  |  |  |  |  |  |  |  |  |  |  |  |  |  |  |  |  |  |  |  |  |  |  |  |  |  |  |  |  |  |  |  |  |  | F30H5.5 |  |
|  |  |  |  |  |  |  |  |  |  |  |  |  |  |  |  |  |  |  |  |  |  |  |  |  |  |  |  |  |  |  |  |  |  |  |  |  |  | F21A3.2 |  |
|  |  |  |  |  |  |  |  |  |  |  |  |  |  |  |  |  |  |  |  |  |  |  |  |  |  |  |  |  |  |  |  |  |  |  |  |  |  | F21A3.4 |  |
|  |  |  |  |  |  |  |  |  |  |  |  |  |  |  |  |  |  |  |  |  |  |  |  |  |  |  |  |  |  |  |  |  |  |  |  |  |  | *set-13* | SET (trithorax/polycomb) domain containing |
|  |  |  |  |  |  |  |  |  |  |  |  |  |  |  |  |  |  |  |  |  |  |  |  |  |  |  |  |  |  |  |  |  |  |  |  |  |  | F38E11.8 |  |
|  |  |  |  |  |  |  |  |  |  |  |  |  |  |  |  |  |  |  |  |  |  |  |  |  |  |  |  |  |  |  |  |  |  |  |  |  |  | *cng-3* | Cyclic Nucleotide Gated channel |
|  |  |  |  |  |  |  |  |  |  |  |  |  |  |  |  |  |  |  |  |  |  |  |  |  |  |  |  |  |  |  |  |  |  |  |  |  |  | Y56A3A.30 |  |
|  |  |  |  |  |  |  |  |  |  |  |  |  |  |  |  |  |  |  |  |  |  |  |  |  |  |  |  |  |  |  |  |  |  |  |  |  |  | T06G6.11 |  |
|  |  |  |  |  |  |  |  |  |  |  |  |  |  |  |  |  |  |  |  |  |  |  |  |  |  |  |  |  |  |  |  |  |  |  |  |  |  | Y62H9A.13 |  |
|  |  |  |  |  |  |  |  |  |  |  |  |  |  |  |  |  |  |  |  |  |  |  |  |  |  |  |  |  |  |  |  |  |  |  |  |  |  | F20D6.8 |  |
|  |  |  |  |  |  |  |  |  |  |  |  |  |  |  |  |  |  |  |  |  |  |  |  |  |  |  |  |  |  |  |  |  |  |  |  |  |  | *aexr-3* | AEX-2 related neuropeptide Receptor |
|  |  |  |  |  |  |  |  |  |  |  |  |  |  |  |  |  |  |  |  |  |  |  |  |  |  |  |  |  |  |  |  |  |  |  |  |  |  | *fbxc-53* | F-box C protein |
|  |  |  |  |  |  |  |  |  |  |  |  |  |  |  |  |  |  |  |  |  |  |  |  |  |  |  |  |  |  |  |  |  |  |  |  |  |  | H10D18.1 |  |
|  |  |  |  |  |  |  |  |  |  |  |  |  |  |  |  |  |  |  |  |  |  |  |  |  |  |  |  |  |  |  |  |  |  |  |  |  |  | *nhr-124* | Nuclear Hormone Receptor family |
|  |  |  |  |  |  |  |  |  |  |  |  |  |  |  |  |  |  |  |  |  |  |  |  |  |  |  |  |  |  |  |  |  |  |  |  |  |  | *pde-5* | PhosphoDiEsterase |
|  |  |  |  |  |  |  |  |  |  |  |  |  |  |  |  |  |  |  |  |  |  |  |  |  |  |  |  |  |  |  |  |  |  |  |  |  |  | *fbxb-114* | F-box B protein |
|  |  |  |  |  |  |  |  |  |  |  |  |  |  |  |  |  |  |  |  |  |  |  |  |  |  |  |  |  |  |  |  |  |  |  |  |  |  | B0294.1 |  |
|  |  |  |  |  |  |  |  |  |  |  |  |  |  |  |  |  |  |  |  |  |  |  |  |  |  |  |  |  |  |  |  |  |  |  |  |  |  | C53C7.3 |  |
|  |  |  |  |  |  |  |  |  |  |  |  |  |  |  |  |  |  |  |  |  |  |  |  |  |  |  |  |  |  |  |  |  |  |  |  |  |  | *srh-200* | Serpentine Receptor, class H |
|  |  |  |  |  |  |  |  |  |  |  |  |  |  |  |  |  |  |  |  |  |  |  |  |  |  |  |  |  |  |  |  |  |  |  |  |  |  | C30E1.2 |  |
|  |  |  |  |  |  |  |  |  |  |  |  |  |  |  |  |  |  |  |  |  |  |  |  |  |  |  |  |  |  |  |  |  |  |  |  |  |  | F23C8.11 |  |
|  |  |  |  |  |  |  |  |  |  |  |  |  |  |  |  |  |  |  |  |  |  |  |  |  |  |  |  |  |  |  |  |  |  |  |  |  |  | *sra-11* | Serpentine Receptor, class A (alpha) |

### Phenotypes enriched

none found

### Anatomy terms enriched

none found

### GO terms enriched

none found

### Expression clusters enriched

|  |  |  |  |
| --- | --- | --- | --- |
| **Group name** | **Number in cluster** | **Enrichment** | **FDR corrected p** |
| Genes that show selective expression in a subset of cell types vs broadly expressed in many cell types. Correspond to 20% - 57% of enriched\_genes for a given cell type. WBPaper00037950:BAG-neuron\_embryo\_SelectivelyEnriched | 6 | 9.19 | 0.0119 |

### Motifs enriched

|  |  |  |  |  |  |
| --- | --- | --- | --- | --- | --- |
| **Motif** | **Logo** | **Possible orthologs** | **Number of motifs in cluster** | **Enrichment** | **FDR corrected p** |
| pTH9385 |  | daf-19 (0.72) | 32 | 2.13 | 0.00017 |
| Max\_Mnt\_SANGER\_5\_FBgn0017578 |  | mxl-1 ref-1 lin-22 aha-1 mxl-2 hlh-30 C27D6.4 | 21 | 2.90 | 0.00046 |
| Dmbx1\_2277 |  | ceh-36 (0.76) alr-1 ceh-53 ceh-45 | 31 | 2.08 | 0.00049 |
| ARNT\_f1 |  | hlh-27 lin-22 aha-1 | 31 | 2.06 | 0.00057 |
| pTH9199 |  | daf-19 (0.72) | 27 | 2.21 | 0.00120 |
| pTH3866 |  | hlh-2 hlh-8 hlh-1 | 32 | 1.92 | 0.00140 |
| Foxg1\_2 |  | lin-31 | 18 | 2.98 | 0.00180 |
| Fer1\_da\_SANGER\_10\_FBgn0037475 |  | lin-32 ceh-32 | 46 | 1.39 | 0.00190 |
| MA0058.2 |  | mxl-1 hlh-30 | 32 | 1.87 | 0.00220 |
| MA0531.1 |  | Y5F2A.4 | 30 | 1.94 | 0.00270 |
| Mw156 |  | mdl-1 aha-1 hlh-30 | 20 | 2.63 | 0.00270 |
| TCF4\_2 |  | hlh-2 | 45 | 1.39 | 0.00370 |
| Fer2\_da\_SANGER\_5\_FBgn0038402 |  | lin-32 | 34 | 1.72 | 0.00500 |
| Mv104 |  | nhr-62 nhr-2 | 46 | 1.34 | 0.00520 |
| V$CREB\_02 |  | crh-1 | 43 | 1.42 | 0.00670 |
| V$GATA1\_03 |  | elt-1 (-0.51) | 27 | 1.97 | 0.00710 |
| RFX4\_1 |  | daf-19 (0.72) | 43 | 1.40 | 0.00950 |
| CREB1\_f1 |  | crh-1 | 44 | 1.37 | 0.01000 |
| pTH9326 |  | nhr-122 (-0.63) | 20 | 2.27 | 0.01500 |
| pTH5005 |  | crh-1 | 31 | 1.70 | 0.01800 |
| pTH8411 |  | tbx-39 | 10 | 3.86 | 0.01800 |
| HIF1A\_si |  | hif-1 (0.62) | 24 | 1.98 | 0.01900 |
| V$E47\_02 |  | hlh-2 | 30 | 1.71 | 0.02100 |
| pTH3037 |  | hlh-15 (-0.51) | 43 | 1.36 | 0.02100 |
| Gmeb1\_1745 |  | attf-1 | 22 | 2.06 | 0.02100 |
| Cutl1\_3494 |  | ceh-44 | 26 | 1.86 | 0.02200 |
| KLF3\_f1 |  | klf-1 | 26 | 1.85 | 0.02400 |
| MA0085.1 |  | lag-1 | 39 | 1.44 | 0.02600 |
| Irx3\_2226 |  | irx-1 | 26 | 1.82 | 0.02800 |
| pTH9211 |  | C01B12.2 | 21 | 2.07 | 0.02800 |
| NDF1\_f1 |  | ngn-1 | 44 | 1.31 | 0.03000 |
| Pax6\_3838 |  | ceh-23 (0.84) alr-1 ceh-14 ceh-45 T13C5.4 | 27 | 1.77 | 0.03100 |
| Eip75B\_SANGER\_5\_FBgn0000568 |  | nhr-118 | 45 | 1.29 | 0.03200 |
| CEBPE\_f1 |  | C48E7.11 | 32 | 1.60 | 0.03300 |
| TBX20\_2 |  | mab-9 | 17 | 2.33 | 0.03300 |
| Hey\_SANGER\_5\_FBgn0027788 |  | lin-22 | 15 | 2.52 | 0.03400 |
| pTH2684 |  | fos-1 | 43 | 1.33 | 0.03500 |
| pTH5805 |  | alr-1 ceh-45 | 19 | 2.15 | 0.03600 |
| ISL1\_f1 |  | lim-7 (0.71) | 25 | 1.82 | 0.03800 |
| pTH9915 |  | zip-3 | 44 | 1.30 | 0.03900 |
| K562\_EGR1\_HudsonAlpha |  | ZC328.2 | 31 | 1.61 | 0.04000 |
| klu\_SANGER\_10\_FBgn0013469 |  | ZC328.2 | 31 | 1.60 | 0.04200 |
| MCR\_f1 |  | nhr-255 | 34 | 1.52 | 0.04500 |
| pTH5781 |  | ceh-32 | 19 | 2.10 | 0.04600 |
| GATA6\_f2 |  | elt-1 (-0.51) | 27 | 1.72 | 0.04600 |
| pTH5033 |  | hlh-8 | 37 | 1.44 | 0.04700 |
| pTH3997 |  | C04F5.9 | 26 | 1.74 | 0.04800 |

### Correlated (and anti-correlated) transcription factors

|  |  |
| --- | --- |
| **Transcription factor** | **Correlation** |
| sox-4 | 0.95 |
| nhr-124 | 0.94 |
| K12H6.12 | 0.93 |
| nhr-258 | 0.92 |
| C06E2.1 | 0.91 |
| nhr-30 | 0.91 |
| nhr-52 | 0.90 |
| nhr-36 | 0.90 |
| nhr-158 | 0.89 |
| nhr-167 | 0.87 |
| nhr-51 | 0.87 |
| dhhc-11 | 0.86 |
| ceh-23 | 0.84 |
| dmd-10 | 0.83 |
| saeg-2 | 0.83 |
| nhr-26 | 0.82 |
| nhr-197 | 0.82 |
| gei-3 | 0.81 |
| ceh-62 | 0.80 |
| nhr-159 | 0.80 |
| nhr-4 | 0.79 |
| ceh-58 | 0.79 |
| nhr-27 | 0.79 |
| mbr-1 | 0.78 |
| nhr-198 | 0.78 |
| lim-6 | -0.41 |
| T22C8.4 | -0.43 |
| vab-15 | -0.44 |
| atf-2 | -0.44 |
| F27D4.4 | -0.45 |
| hlh-2 | -0.46 |
| mab-9 | -0.47 |
| nhr-9 | -0.47 |
| irx-1 | -0.48 |
| cnd-1 | -0.48 |
| ceh-13 | -0.48 |
| php-3 | -0.49 |
| dmd-3 | -0.50 |
| hlh-15 | -0.51 |
| elt-1 | -0.51 |
| nhr-13 | -0.53 |
| hlh-32 | -0.55 |
| unc-62 | -0.56 |
| bar-1 | -0.56 |
| nob-1 | -0.57 |
| mab-5 | -0.58 |
| lin-39 | -0.61 |
| nhr-122 | -0.63 |
| tlp-1 | -0.64 |
| pal-1 | -0.64 |

### ChIP peaks enriched

none found
